# Economic impact of multiple recurrent *Clostridioides difficile* infection in a community teaching hospital

**DOI:** 10.1017/ice.2025.10295

**Published:** 2025-11

**Authors:** Joseph Reilly, Gemma Downham, Manish Trivedi

**Affiliations:** 1 Department of Clinical Pharmacy, AtlantiCare Regional Medical Center, Pomona, NJ, USA; 2 Department of Patient Safety and Infection Prevention, AtlantiCare Regional Medical Center, Pomona, NJ, USA; 3 Department of Infectious Diseases, AtlantiCare Regional Medical Center, Pomona, NJ, USA

## Abstract

A retrospective study evaluating the economic impact on a community teaching hospital of 29 patients with multiple *Clostridioides difficile* infection admissions in a 4-year period showed an estimated net loss of $2,232,997, posing a substantial economic impact on the hospital.

## Introduction

*Clostridioides difficile* infection (CDI) is a major burden to patients and the healthcare system, with approximately 500,000 cases per year in the United States and 30,000 annual deaths directly attributable to CDI.^1–3^

Roughly 20–35% of patients with an initial CDI case experience a recurrence, and 50–65% of patients who have a first recurrent CDI (rCDI) case experience a second or subsequent rCDI.^3^ Rates of rCDI have been increasing in recent years, and the disease is considered a major public health threat.^3,4^ Multiple recurrent CDI (mrCDI; ≥2 rCDI cases) poses a significant burden.^5^ Compared to an initial CDI case, healthcare costs for rCDI are notably higher, with an additional all-cause economic burden of $15,000 to $60,000.^6^

There are few data available on the net economic impact of repeat CDI admissions at the level of a single hospital or healthcare system. This study evaluated the economic impact on a community teaching hospital of repeat admission of patients with multiple CDI admissions.

## Methods

### Study design

A retrospective cohort study of patient medical records at AtlantiCare Regional Medical Center (Pomona, NJ) was conducted for the period of January 2017 through December 2020, using clinical software (TheraDoc®, Premier Inc.). This study was approved by the AtlantiCare Regional Medical Center Institutional Review Board.

### Eligibility criteria

Patients were included if they had ≥3 inpatient admissions due to CDI (primary diagnosis) and tested positive for CDI between January 2017 and December 2020. Admissions for CDI occurring ≥ 8 weeks after the completion of treatment for a previous CDI case were included to provide a real-world assessment of disease impact in a clinical setting, regardless of whether it is defined as “recurrence” or “reinfection” by standard definitions. CDI was identified by two positive sequential tests: a nucleic acid amplification test for toxigenic strains of *C. difficile* and an enzyme immunoassay for glutamate dehydrogenase. Patients who tested positive for CDI but did not display clinical signs and symptoms of infection were excluded. Admissions for reasons other than CDI were excluded.

### Data extraction

Information collected included: demographics; comorbid conditions and immune status; length of hospital stay for each CDI-related admission; acuity of care; CDI severity; mortality; utilization of antimicrobials; laboratory parameters; and time between admissions. Sepsis was recorded in the patient’s medical record and determined by hospital criteria including one sign of organ dysfunction and ≥ 2 indicators for systemic inflammatory response syndrome.

Immunocompromised was defined as: diagnosis of HIV/AIDS; receiving chemotherapy or radiation therapy (concomitant or within 3 months of admission); prior organ transplant; chronic corticosteroid use (≥10 mg daily prednisone or equivalent); autoimmune disorder; or treatment with an immune suppressing medication.

For CDI severity, non-fulminant, non-severe disease was defined as concomitant WBC count <15,000 cells/mL and serum creatinine (sCr) <1.5 mg/dL. Non-fulminant, severe disease was concomitant WBC count ≥15,000 cells/mL and sCr ≥1.5 mg/dL. Fulminant disease was the presence of hypotension or shock, ileus, or megacolon.^7,8^

### Patient data analysis

Investigators reviewed the extracted data and assessed for factors potentially contributing to developing CDI. Demographic characteristics and healthcare utilization were summarized with descriptive statistics.

### Economic analysis

The net economic impact of CDI-related hospitalizations for multiple CDI was estimated for the hospital. The cost of one inpatient day in 2020 at our non-profit hospital in Southern New Jersey was set at $3200.^9^ The cost of one day in the intensive care unit (ICU) was set at $5000 (adjusting available figures from 2010 to 2020 using the Medical Care component of the Consumer Price Index).^10,11^ The estimated reimbursement for a hospitalization with a primary diagnosis code of CDI (ICD9-CM code: 008.45; ICD 10-CM codes: A04.7, A04.71 or A04.72) was set at $8229 for non-fulminant cases and $15,670 for fulminant CDI cases, taking into account published literature, Centers for Medicare and Medicaid reimbursement rates, and current hospital data.^12,13^ Costs were adjusted to 2020 US dollars.

## Results

### Demographics and baseline characteristics

Twenty-nine patients were included in this analysis, with a mean (SD) age of 58.9 (14.3) years; 55% of patients were female (Table 1). All 29 patients received CDI-related antimicrobials (63.9% received oral vancomycin, 36.1% received fidaxomicin) during admission(s). Sepsis was reported during 36 admissions (33.3%), and 31 (28.7%) were fulminant CDI cases. Many patients had ≥1 comorbid conditions that are known risk factors for (r)CDI (Table 1).

**Table 1.** Patient characteristics

	All patients (*N* = 29)
Age, years; median (range)	57 (27–94)
Female; n (%)	16 (55.2)
White race; n, (%)	16 (55.2)
BMI ≥ 30 kg/m^2^; n (%)	11 (37.9)
Admitted from home; n (%)	24 (82.8)
Admitted from long-term care facility; n (%)	5 (17.2)
Medical history, n (%)	
History of gastrointestinal surgery	13 (44.8)
Chronic kidney disease	11 (37.9)
Immunocompromising condition	10 (34.5)
Inflammatory bowel disease	3 (10.3)
Cirrhosis	4 (13.8)
Duration between CDI-related admissions, days; median (range)	49 (14–532)

BMI, body mass index

### Healthcare utilization

A total of 108 admissions due to CDI in 29 patients were identified over the study period of 4 years; 1006 inpatient hospital days were attributed to a CDI diagnosis in this sample, including 74 days in the ICU. Five additional CDI cases were excluded from the analysis because CDI developed during a hospitalization and not as an admitting diagnosis.

Patients had a median (range) number of admissions of 3 (3–6) per patient. The median (range) length of stay per admission was 7 (1–69) days. The median (range) duration between admissions was 49 (14–532) days. Most admissions were for CDI recurring within 8 weeks of the previous case. In addition, all 29 patients in the sample had ≥2 non-CDI-related admissions during the study period, which totaled 78 non-CDI-related admissions.

### Economic outcomes

Total hospitalization costs for the 29 patients over the 4-year study period were estimated to be $3,352,400, attributed to 108 CDI admissions (primary diagnosis code). The estimated total reimbursement for 108 CDI-related hospitalizations was $1,119,403.

Thus, we estimated that the community teaching hospital had a net loss of $2,232,997 over the 4-year period due to CDI-related hospitalizations. This translates to a net loss per patient of approximately $77,000 over the 4-year study period.

### Patient outcomes

One patient died within the study period, due to respiratory failure, with hepatic encephalopathy, colitis, and toxic megacolon.

## Discussion

Few data exist on multiple CDI-attributable hospitalization costs and resource utilization. A single-center study estimated the hospitalization costs for rCDI-related admissions to be $30,122 per patient in the 1-year study period (2016 US dollars).^14^ More recent estimates from a systematic literature review and a component-based cost synthesis estimated rCDI-attributable hospitalization costs to be an average of $45,000–$52,000 per patient per year.^3^ Here, we estimated an average cost of multiple CDI-related hospitalizations to be $28,900 per patient, per year. Our economic analysis for CDI may underestimate the true financial losses incurred that are attributable to multiple CDI. Full reimbursement for every CDI admission was assumed, which may not be the case with multiple CDI if a readmission occurs within 30 days of the previous discharge.

All patients in this group had ≥2 non-CDI–related admissions during the study. These admissions pose greater possible financial losses for the hospital.

Limitations of this analysis include small sample size, lack of a comparator group, findings that may not be generalizable to other types of hospitals or healthcare systems, and outcomes that may differ for payors other than Medicare, which may have higher reimbursement rates for hospitalizations with a primary diagnosis of CDI that could result in an underestimate of the reimbursement and an overestimate of the net losses in this analysis. The more flexible definition of multiple CDI cases used in this manuscript may overestimate the economic impact of rCDI.

Our findings indicate that there is an ongoing cycle of CDI admissions among a subset of patients who experience CDI, which poses a substantial economic impact on the hospital.
